# Cross-Sectional Associations Between Wake-Time Movement Compositions and Mental Health in Preschool Children With and Without Motor Coordination Problems

**DOI:** 10.3389/fped.2021.752333

**Published:** 2021-11-30

**Authors:** Denver M. Y. Brown, Matthew Y. W. Kwan, Sara King-Dowling, John Cairney

**Affiliations:** ^1^Department of Psychology, University of Texas at San Antonio, San Antonio, TX, United States; ^2^Infant, Child and Youth Health (INCH) Lab, Department of Family Medicine, McMaster University, Hamilton, ON, Canada; ^3^Department of Child and Youth Studies, Brock University, St. Catherines, ON, Canada; ^4^Division of Oncology, The Children's Hospital of Philadelphia, Philadelphia, PA, United States; ^5^School of Human Movement and Nutrition Sciences, University of Queensland, Brisbane, QLD, Australia

**Keywords:** physical activity, sedentary behavior, daily time use, mental ill-being, developmental coordination disorder

## Abstract

Movement behaviors have been found to be important correlates of health for children and may be particularly important for children with Developmental Coordination Disorder (DCD) who often experience greater mental health problems. To date, however, little research has investigated the daily movement composition of preschool children with Developmental Coordination Disorder (DCD) and/or its association with mental health. The purpose of the current study was to: (1) examine whether differences in movement compositions (i.e., sedentary time, light physical activity, moderate-to-vigorous physical activity) exist between typically developing (TD) preschool-age children and those at risk for DCD (rDCD); and (2) investigate associations between movement compositions and mental health indicators. This cross-sectional study used the baseline cohort data from the Coordination and Activity Tracking in CHildren (CATCH) study. A total of 589 preschool-age children (Mage = 4.94 ± 0.59 years; 57.4% boys) were included in this analysis, of which 288 scored at or below the 16th percentile on the Movement Assessment Battery for Children-2 and were thus classified as rDCD. Wake time movement behaviors were measured using accelerometers and parents completed the Child Behavior Checklist to assess their child's mental health (i.e., internalizing and externalizing problems). Compositional data analysis techniques were used. After adjusting for potential confounders, the results demonstrated similar movement compositions between TD and rDCD children. Among the full sample, findings revealed a significant association between sedentary time and externalizing problems, however, each of the other associations did not reach statistical significance. These results are consistent with emerging evidence demonstrating similar patterns of physical activity and sedentary time among TD children and those classified as rDCD during the preschool years. Although movement behaviors explained little variance in mental health during this period, future research should investigate when movement compositions diverge, and how these changes may impact the mental health of TD children and those classified as rDCD later in childhood.

## Introduction

An abundant body of literature has demonstrated the positive impact of moderate-to-vigorous physical activity (MVPA) for fitness, motor and cognitive development as well as psychosocial, skeletal and cardiometabolic health in early childhood (1). However, much of this work has failed to take into account that other movement behaviors—such as sleep, sedentary time and light physical activity (LPA)—also play an important role (2). This constrained approach has sparked a push for the adoption and application of the 24-h movement paradigm to better understand how interrelationships among movement behaviors across the whole day affect healthy development during the early years (3, 4). Previous work has shown that increasingly sedentary patterns of movement behaviors begin to emerge as early as the preschool years (5–8), resulting in less favorable health outcomes (5), which can track into adulthood (9). Therefore, it is imperative that we improve our current understanding of the interactive nature of movement behaviors and their relationship with mental health so that we can design effective interventions moving forward.

Certain populations such as children with neurodevelopmental disorders experience a variety of barriers and limitations to daily activities that may cause them to engage in less active and more sedentary lifestyles than their typically developing (TD) peers (10, 11). This is of particular concern given that this population is at an increased risk of physical comorbidities (12). Developmental Coordination Disorder (DCD) is a neurodevelopmental disorder that affects ~2–6% of children (13, 14) and has received considerable attention regarding patterns of movement behaviors during childhood, but work in this area has focused largely on MVPA. For instance, a systematic review of the literature found children with DCD engage in lower levels of MVPA than TD children and these differences persist as they age into adolescence (15). While there has been little research on the early years, recent work using accelerometry suggests that preschool-aged children at risk for DCD (rDCD) engage in similar amounts of MVPA, LPA and sedentary time compared to their TD peers (16). However, studies taking a more comprehensive approach that moves beyond considering absolute amounts of time engaging in each behavior to instead focus on the relative proportion of time spent engaging in each behavior within a finite window have yet to be conducted.

To date, very few studies have taken an integrative approach to investigate associations between movement behaviors and health outcomes among preschool children. Even fewer studies have adopted compositional data analysis methods to examine how time spent engaging in one movement behavior relative to the others or reallocation of time between movement behaviors is related to healthy development. Recent work using accelerometry has shown that 24-h movement compositions significantly predict fundamental motor skills (17, 18) and cognitive development (17, 19) in preschool-age children. Although evidence has demonstrated beneficial associations between different intensities of physical activity and indicators of mental health within samples of children and youth between the ages of 6–17 years (20–22), the only study to examine these relationships among preschool-age children observed null relationships (17). Combined with the fact that children at risk for DCD experience higher rates of mental health problems beginning as early as the preschool years (23), this dearth of research highlights the need for additional work—particularly among larger and more diverse samples—to better understand whether movement behaviors influence mental health in the early years. Although existing literature has examined different pathways by which physical activity and sedentary time influence indicators of mental health among children classified as rDCD (24, 25), studies adopting novel techniques to investigate how the interplay between these movement behaviors affects their mental health have yet to be conducted.

Therefore, the purpose of the present study is two fold: (1) to examine whether differences in wake-time movement compositions (i.e., sedentary time, LPA, MVPA) exist between preschool children classified as TD and rDCD; and (2) to investigate associations between the movement composition of preschool children and indicators of mental health.

## Methods

### Participants

The present study is cross-sectional, utilizing baseline cohort data from the Coordination and Activity Tracking in CHildren (CATCH) study. This on-going prospective cohort study is being undertaken in Southeastern Ontario, Canada. The purpose of the CATCH study is to examine relationships among motor coordination, physical fitness, physical activity, and health outcomes beginning in early childhood (26). A rolling recruitment between October 2013 and June 2017 was used to assemble the cohort. Families and children began attending the Infant, Child and Youth Health Lab at McMaster University for baseline assessments in February 2014. Children 4–5 years of age were screened for poor motor coordination during several stages of the recruitment process to achieve a target sample size of 300 TD children and 300 children classified as rDCD. Children with a physical disability or a medical condition that affected their motor coordination (e.g., cerebral palsy) were not eligible for the study, in addition to children with a birth weight lower than 1,500 g, to rule out conditions other than DCD that may account for children's poor motor coordination. Additional details of the study design and recruitment procedures are available elsewhere (26, 27). Informed, written consent was provided by the parents/guardians of all participants. The CATCH study protocol was approved by an institutional research ethics board.

### Measures

#### Demographics

Parents reported their total household income as well as their child's race/ethnicity and sex. Total household income was coded into four groups: <$50,000, between $50,000 and $99,999, between $100,000 and $149,999, and >$150,000. Race/ethnicity was coded into two groups: White, and non-White, which included Asian, Middle Eastern, Black, Indigenous, Latino and Mixed/Other.

#### Anthropometric Data

Height and weight were obtained using a calibrated weigh scale and stadiometer and used to calculate body mass index [mass (kg)/height (m)^2^] *z*-scores based on age and sex as per the Center for Disease Control growth charts (28).

#### Developmental Coordination Disorder Risk

The Movement Assessment Battery for Children 2nd Edition (MABC-2) is a standardized test consisting of 8 items categorized into 3 groups: (1) manual dexterity, (2) aiming and catching, and (3) static and dynamic balance. Children's raw scores on these items are converted into standardized scores based on age and then further classified into overall percentile ranks. The MABC-2 is widely used for the identification of DCD and has been shown to be valid and reliable for assessing poor motor coordination in children ages 3 to 5 years (29). The tool has also been identified as the criterion standard for assessing DCD in children (30). In the current study, children who scored above the 16th percentile were classified as TD, and children who scored at or below the 16th percentile were classified as at risk for DCD (31). All MABC-2 assessments were administered and scored by trained research assistants. As noted earlier, the design of the CATCH study also involved exclusion criteria pertaining to conditions that may otherwise explain a child's poor motor coordination and thus result in them being classified as rDCD for extraneous reasons.

#### Movement Behaviors

Accelerometry is a well-established device-assessed measure of physical activity and sedentary time in children (32). Children's wake-time movement behaviors—sedentary time, LPA, and MVPA—were assessed using an Actigraph GT3X+ activity monitor device worn on their right hip secured by a belt around their waist for 7 days. The device was only removed for periods of sleep time and prolonged water exposure. Parents were asked to keep a diary to record when the accelerometer was put on and taken off. Minimum wear time criteria for including a child's accelerometer data were set at 3 days with a minimum of 10 h per day wear time (33). The average daily wake-time movement composition across the whole measurement period was used in our analyses. Given that young children are inclined toward short bursts of activity, data were analyzed in three-second epochs using ActiLife 6 software (ActiGraph, Pensacola, FL) to allow for a more accurate representation of activity levels (34–36). The 2008 Evenson activity cut point for children was used to determine time spent in sedentary behavior (in minutes per day), as well as time spent engaging in light, and moderate-to-vigorous physical activity (in minutes per day). These cut points have been previously validated in children ages 5–8 (37).

#### Internalizing and Externalizing Problems

Parents completed the Child Behavior Checklist (CBCL) for ages 1.5–5 years during baseline testing for the CATCH study (38). The CBCL is a validated and reliable tool commonly used to assess externalizing and internalizing problems, among children. The test-retest reliability of the CBCL scale scores range from 0.68 to 0.92 and reported classification accuracy has been shown to range from 74 to 84% (39). In the present study, we used the DSM-IV broad band internalizing and externalizing problems scales. These scales have been used previously with children at risk for DCD (40). The internalizing problems scale score represents the sum of the emotionally reactive, anxious/depressed, withdrawn, and somatic complaints syndrome subscales, whereas the externalizing problems scale score represents the attention problems and aggressive behavior syndrome subscales. High levels of validity and reliability of the DSM-IV oriented scales have been established among clinical and non-clinical samples (41), and extensive normative data are available for children between the ages of 1.5–5 years. Internal consistency for the DSM-IV orientated scales has been reported at 0.75, with a test-retest reliability of 0.83 (41). Although DSM-V oriented scales have since been created, the values for these scales were considerably right skewed, and various transformations did not improve the distribution of the data; therefore, the standardized t-score DSM-IV scales were used. The CBCL DSM-IV standardized t-scores represent a comparison of a child's raw score to what would be a typical score reported for their gender and age based on a normative sample. These scores are scaled so that 50 is the mean for the child's age and gender, with a standard deviation of 10 points and higher scores representing greater problems.

### Data Analysis

All analyses were performed in R (Version 4.0.3) and R Studio (Version 1.3.1093). First, we inspected the data for missingness using the *mice* package (42). Data were considered missing at random and multiple imputation by chained equations was conducted using the *mice* package to replace missing values. However, the packages used for subsequent compositional analyses cannot handle multiply imputed datasets, and therefore, one of the five imputed datasets was randomly selected for the purpose of our analyses. When multiple imputation and full information maximum likelihood cannot be implemented to handle missing data, single stochastic regression imputation (i.e., using only one of *N* multiply imputed datasets) is considered the next best alternative over other procedures such as listwise deletion and mean imputation because it models in random error to obtain more realistic values, resulting in a relatively unbiased estimate in large samples (43).

Next, we conducted the primary data analyses, which consisted of (1) comparing wake-time movement compositions between children classified as TD and rDCD, and (2) investigating the relationship between the movement composition and indicators of mental health, and the influence that changes to the movement composition may impart for mental health among preschool children.

The *compositions* (44), and *deltacomp* (45) packages were used for all compositional data analyses. Wake-time movement behaviors (sedentary time, LPA, MVPA) assessed via device-based measures such as accelerometry are mutually exclusive and exhaustive parts of the movement composition. Collectively, time spent in each of these behaviors must sum to the entire wake-time period over the course of a day. Simply stated, the amount of time an individual engages in one movement behavior cannot be changed without an equivalent change among the other behaviors. For example, reducing sedentary time would mean an individual spends more time engaging in LPA or MVPA. Compared to previous work that has commonly reported movement behaviors such as physical activity in absolute terms, movement compositions are relative data and therefore take into account the codependence amongst all behaviors that make up the composition. Due to potential issues with multicollinearity, however, there are statistical challenges with including all movement behaviors within a single model using traditional statistical methodologies (46). Compositional data analysis techniques can be used to address this limitation (47). Compositional data analysis involves expressing time spent engaging in different movement behaviors during a finite period in relative terms, as a set of isometric log-ratio (ilr) coordinates (48).

Given that ilrs cannot be computed if there are zeros in the data that represents the relative time spent in each movement behavior, the first step in our analysis involved checking for zero values across the three parts (sedentary time, LPA, MVPA) of our daily wake-time composition. Once we confirmed none were present, we created the ilr coordinates using a sequential binary partition process (49). Computing the first pivot coordinate to provide an ilr involved partitioning the composition by coding time spent in one behavior as the numerator (+1) in the equation, and time amongst the other two behaviors coded as the denominator (−1). After partitioning out the first behavior, it is coded as an uninvolved part (0) in the sequential binary partition when creating the second pivot coordinate. Time spent engaging in the remaining two behaviors were then coded to be in the numerator (+1) and the denominator (−1). The pivot coordinates were used to produce the ilrs that express the movement composition. To illustrate this process with an example, the first coordinate may include all relative information regarding sedentary time vs. the geometric mean of time spent engaging in any intensity of physical activity, and the second coordinate may represent LPA vs. MVPA. Following this example, ilr transformations of the movement behavior composition were expressed using the following equations:


(1) $$\begin{array}{r} ilr_{1}^{\left(SB\right)}=\sqrt{\frac{2}{3}}\ln\frac{SB}{\sqrt{LIPA\;\times \;MVPA}} \end{array}$$

(2) $$\begin{array}{r} {\;ilr}_{2}^{\left(SB\right)}=\frac{1}{\sqrt{2}}\ln\frac{LIPA}{MVPA} \end{array}$$

The final ilrs provided geometric means of all parts, which were linearly adjusted to sum to 1, or 100% of the wake-time period, with each movement behavior (i.e., part) expressed as a proportion of the whole composition. We closed our composition to 724 min (~12 h), which represented the mean daily wear time for the sample. Given that wear time varies across participants, this means that the amount of time each participant spent engaging in each movement behavior was adjusted to a wake-time window of 724 min.

To answer the first research question of whether differences in mean time-use movement compositions exist between preschool children classified as TD and rDCD, a compositional multivariate analysis of covariance model was computed. Wake time movement behaviors (sedentary time, LPA, MVPA) were included as the primary variables of interest, rDCD status (yes/no) was set as the grouping variable, and the model was adjusted for covariates (age, sex, socioeconomic status, ethnicity) known to be related to physical activity behavior (50). Models with each movement behavior set as the dominant activity in the model provide equivalent estimates, therefore, only one set of ilr coordinates were constructed, with sedentary time set as the dominant activity.

For the second research question regarding whether movement compositions impact indicators of mental health among preschool children, we pooled the subsamples of children with TD and rDCD given the similarity in their movement compositions. Three sets of ilrs were constructed, with each set treating a different movement behavior as the primary variable of interest. The ilrs were used to compute a series of linear regression models examining associations between each movement behavior and the indicators of mental health. The regression coefficients and standard errors for the first ilr coordinate for sedentary time, LPA, and MVPA are presented, considering the focus is on time spent in each behavior relative to the remaining two behaviors. Each model was adjusted for rDCD status, age, sex, socioeconomic status, race/ethnicity and body mass index percentile. Assumptions for linearity, homogeneity and normality were examined using the *performance* package (51). All assumptions were satisfied for each model, although a total of 27 (4.6%) and 24 (4.1%) influential observations were identified for the internalizing and externalizing problems models, respectively, as per Cook's distance values >4/*n*. Ordinary least squares regression estimates are sensitive to highly influential observations, therefore, robust regression was employed using the *robustbase* package to reduce the influence of these observations (52).

Finally, compositional isotemporal substitution modeling was performed to examine the impact on internalizing and externalizing problems of reallocating 5, 10, 15, 20 and 25 min in one movement behavior to another (e.g., replacing 5 min of sedentary time with 5 min of LPA). These models followed the methods outlined by Dumuid et al. (53) and were computed using the *deltacomp* package (45). This analysis involved creating a series of new movement compositions to reflect the range of 5 min isotemporal substitution increments for each movement behavior contrast (sedentary time vs. LPA; sedentary time vs. MVPA; LPA vs. MVPA), with the base regression model set as the starting composition. Statistical significance for all analyses was set at α < 0.05.

## Results

### Data Inspection

Inspection of the data revealed 10 (1.7%) missing values for ethnicity, 17 (2.9%) for total household income, and one (0.2%) for the MABC-2. A total of 75 participants (12.7%) had missing accelerometry data, and seven participants (1.2%) had missing mental health data. Participants with missing accelerometry data had significantly higher body mass index percentile values as well as lower household income and MABC-2 scores. Participants with missing mental health data had significantly lower household income. For these reasons, data was considered missing at random and missing values were replaced via stochastic regression imputation.

### Sample Demographics

On average, participants in the present study were 4.94 years of age and the sample consisted of slightly more than half boys (57.4%), were identified primarily as White (81.0%) and lived in households with a total income of more than $50,000 (86.8%). There were more children identified as typically developing (*n* = 301, 51.1%) than those classified as rDCD (*n* = 288, 48.9%). Descriptive statistics for demographic variables, indicators of mental health as well as absolute and relative time spent engaging in each movement behavior are presented for the full sample, and by group, in Table 1.

**Table 1:** Descriptive statistics for demographic, behavioral, and mental health variables.

	**Total sample (*N* = 589)**	**TD children (*n* = 301)**	**rDCD children (*n* = 288)**
Age	4.94 (0.59)	5.00 (0.61)	4.87 (0.56)
Sex (male)	338 (57.4)	145 (48.2)	193 (67.0)
BMI *z*-score	56.07 (27.14)	54.29 (27.23)	57.93 (26.96)
**Household income**			
<$50,000	78 (13.2)	35 (11.6)	43 (14.9)
$50,000–$99,999	178 (30.2)	90 (29.9)	88 (30.6)
$100,00–$150,000	184 (31.2)	88 (29.2)	96 (33.3)
≥$150,000	149 (25.3)	88 (29.2)	61 (21.2)
**Race/ethnicity**			
White	477 (81.0)	238 (79.1)	239 (83.0)
Asian	36 (6.1)	17 (5.6)	19 (6.6)
Middle Eastern	7 (1.2)	4 (1.3)	3 (1.0)
Black	12 (2.0)	11 (3.7)	1 (0.3)
Indigenous	3 (0.5)	3 (1.0)	0 (0.0)
Latino	5 (0.8)	1 (0.3)	4 (1.4)
Mixed/other	49 (8.3)	27 (9.0)	22 (7.6)
MABC-2 percentile	33.17 (29.12)	56.18 (23.40)	9.13 (5.48)
**Absolute accelerometer measured time-use (min/day)**bb			
Sedentary time	451.24 (45.55)	451.69 (44.30)	450.78 (46.90)
LPA	200.90 (27.83)	199.75 (28.38)	202.10 (27.25)
MVPA	71.81 (19.66)	71.97 (20.85)	71.63 (18.38)
Total wear time	723.92 (41.72)	723.34 (40.95)	724.54 (42.58)
**Mean time-use composition (% of wear time)**			
Sedentary behavior	62.33 (5.16)	62.47 (5.30)	62.19 (5.02)
LPA	27.75 (3.50)	27.61 (3.54)	27.90 (3.46)
MVPA	9.92 (2.65)	9.94 (2.78)	9.90 (2.52)
Internalizing problems	48.02 (9.82)	46.52 (9.08)	49.60 (10.33)
Externalizing problems	45.55 (9.27)	43.58 (8.44)	47.61 (9.66)

*LPA, light physical activity; MVPA, moderate-to-vigorous physical activity. Values in table represent means with standard deviations in parentheses or total cases with percent in parentheses*.

### Group Differences in Movement Compositions Based on rDCD Status

**Figure 1:**
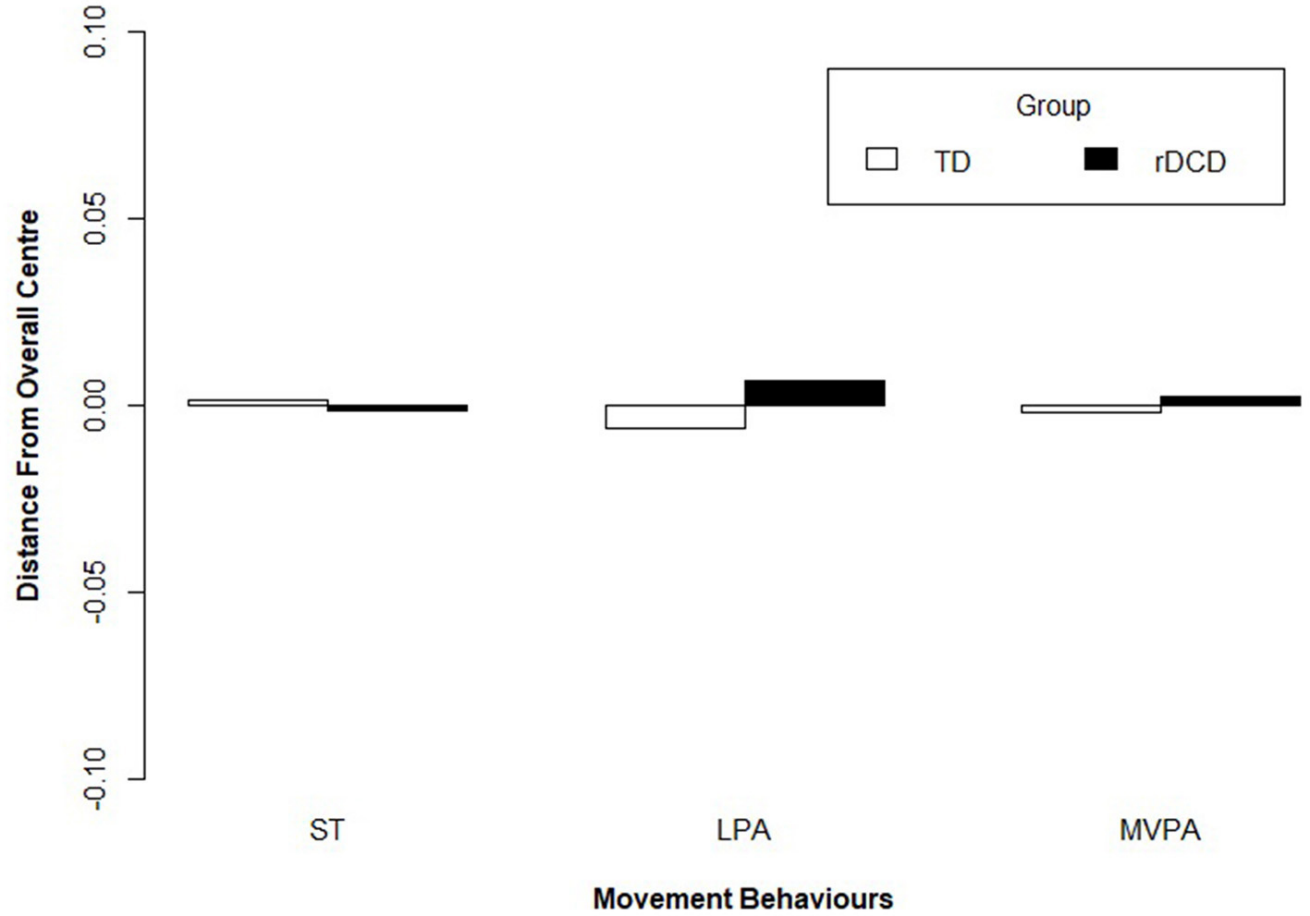
Compositional analysis of the mean time spent in sedentary time (ST), light physical activity (LPA) and moderate-to-vigorous physical activity (MVPA) with respect to the overall mean time composition, by group.

**Figure 2:**
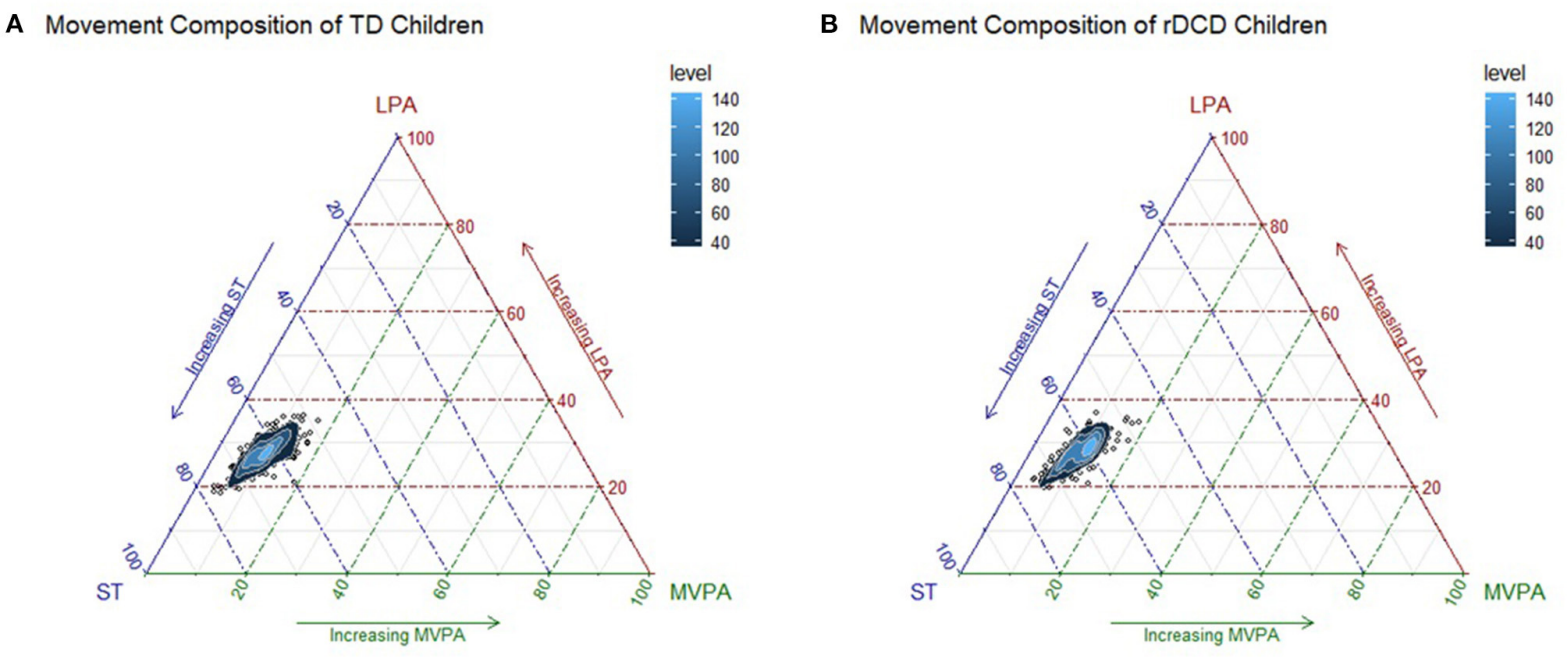
Ternary plots of the sample compositions of time spent in sedentary time (ST), light physical activity (LPA) and moderate-to-vigorous physical activity (MVPA), for TD **(A)** and rDCD children **(B)** with 2D kernel density estimations.

The results of our compositional multivariate analysis of covariance testing whether movement compositions differed between preschool children classified as TD and rDCD failed to reveal significant differences in their mean daily time use patterns after adjusting for covariates, *F*_(2,580)_ = 0.56, *p* = 0.57, Λ = 1.00. The relative distribution of time spent engaging in sedentary time, LPA and MVPA for each group is presented in Figure 1 as the log-ratio differences between the group compositional mean and the full sample compositional mean after centering the data. The scatterplot distributions of the group compositions are shown in separate ternary diagrams (Figures 2A,B).

### Associations Between Movement Compositions and Indicators of Mental Health

Given the similarity in movement compositions for TD and rDCD children, we pooled the groups and investigated associations with mental health using the full sample. The results of our regression models demonstrated preschool-age children's movement compositions explained 3% of the variance for internalizing problems (Adjusted *R*^2^ = 0.03) and 8% for externalizing problems (Adjusted *R*^2^ = 0.08). Within the compositional linear regression models, a significant negative association was found when predicting externalizing problems from sedentary time (*p* = 0.03), however, significant associations were not observed for the other five relationships (all *p*'s > 0.05; Table 2).

**Table 2:** Associations between movement compositions and indicators of mental health.

	**Indicators of mental health**			
	**Internalizing problems**		**Externalizing problems**	
	***B* (SE)**	***p-*value**	***B* (SE)**	***p-*value**
Sedentary behavior	−0.26 (2.59)	0.92	**−5.24 (2.44)**	**0.03**
Light physical activity	3.08 (3.68)	0.40	4.16 (3.64)	0.25
Moderate-to-vigorous physical activity	−2.82 (2.10)	0.18	1.08 (2.15)	0.62

*Significant associations are bolded*.

**Figure 3:**
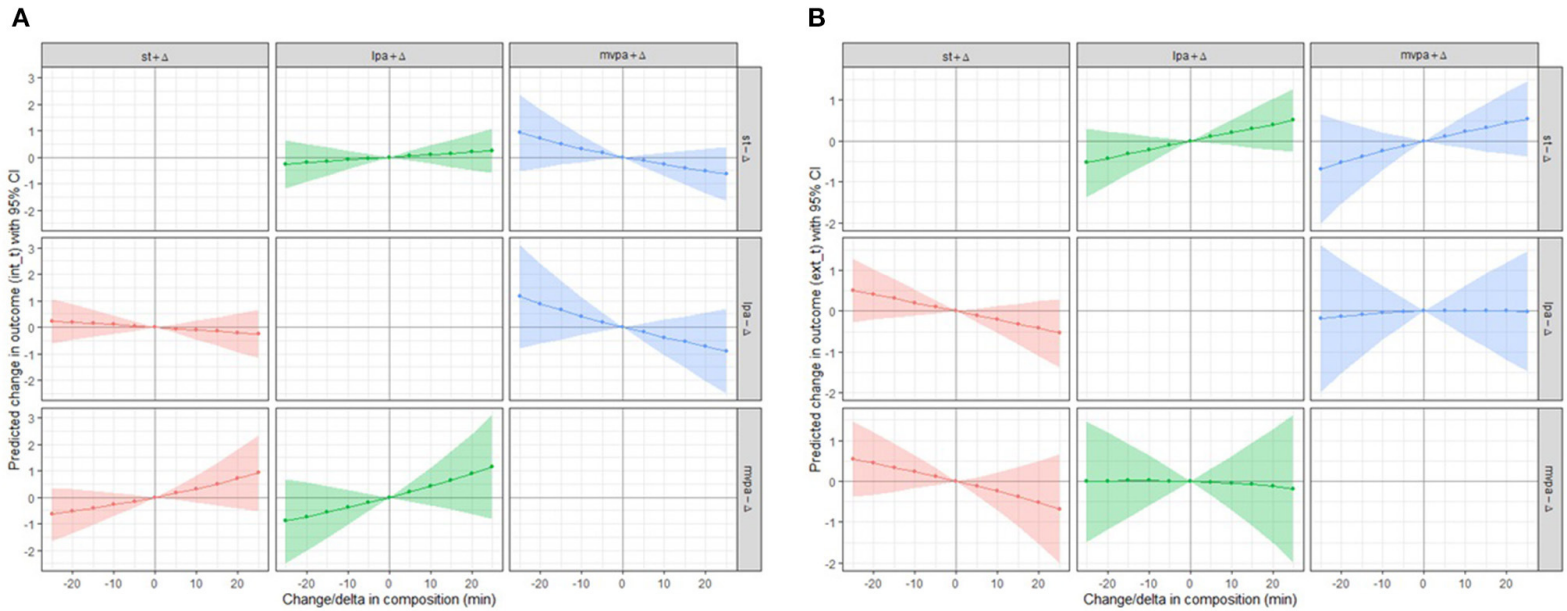
The impact of reallocating time (5 to 25 min) between movement behaviors (e.g., replacing 25 min of sedentary behavior with 25 min of light physical activity, while holding moderate-to-vigorous physical activity constant) on internalizing problems **(A)** and externalizing problems **(B)**.

Isotemporal substitution modeling results are presented graphically in Figure 3. Reallocating 5, 10, 15, 20, and 25 min of time between each of the movement behaviors was not found to impact internalizing problems (all *p*'s > 0.05). This was to be expected given the base regression model failed to show a significant association between any one movement behavior (relative to the others) and internalizing problems. Contrary to our base regression model, we failed to observe a significant influence on externalizing problems when time spent engaging in any one movement behavior was replaced with 5, 10, 15, 20, and 25 min of time engaging in another movement behavior (all *p*'s > 0.05).

## Discussion

This study was the first to examine whether wake-time movement compositions differ between preschool children classified as TD and rDCD, and represents the largest study to investigate the impact of wake-time movement behaviors on indicators of mental health among preschool children using accelerometry. Our results indicated that movement compositions were relatively similar for TD preschool-age children and those classified as rDCD; with both groups, on average, engaging in nearly 5 h of activity of varying intensities during a 12-h wake period. Given the similarity in movement compositions between TD children and those classified as rDCD, we pooled the sample and adjusted for rDCD status when investigating associations between sedentary time, LPA and MVPA with indicators of mental health. Findings demonstrated a significant negative relationship for sedentary time (relative to time in LPA and MVPA) and externalizing problems, whereas null associations were observed for each of the other relationships. Collectively, these findings suggest preschool children engage in a considerable amount of activity across the course of a day, but their movement behavior patterns explain very little variance in parent-reported measures of internalizing and externalizing problems.

Previous research has generally suggested that as children and adolescents with DCD grow older, they engage in increasingly less active and more sedentary lifestyles than their TD peers (15). This study was the first to use compositional data analysis techniques to determine whether these traditionally observed differences in movement behaviors are apparent as early as the preschool years. Using accelerometry, our findings showed that the relative amount of time spent engaging in sedentary time, LPA and MVPA across the course of an average day was roughly equivalent for TD children and those classified as rDCD. These movement compositions involved a smaller portion of time spent engaging in physical activity and more time in sedentary behavior compared to previous work investigating Canadian preschool children (17), but were similar to movement compositions that have been reported by two studies conducted in Brazil (18, 19). Although the present study focused on general patterns of movement behaviors, other work using the baseline CATCH cohort suggests that there may be subtle differences in how preschool children classified as rDCD accumulate their MVPA (16). Given that the CATCH study will track this sample into late childhood, future work will provide a better understanding of when differences in movement compositions begin to manifest. For instance, it is reasonable to hypothesize that children classified as rDCD will become less active and more sedentary when they reach middle childhood, as this period represents when many children begin playing organized sports and engaging in other active pursuits that require greater motor demands. For children with DCD, their poor motor skills may affect their confidence to participate (54), thus leading them to engage in more sedentary pursuits. Fortunately, multiple waves of data from the CATCH study will be able to answer many of the outstanding questions in this area.

Beyond examining potential differences in movement compositions among children classified as TD and rDCD during the preschool years, the present study also contributes to the dearth of literature investigating the impact of movement behaviors on mental health during the early years. For the most part, our findings align with previous research that has failed to demonstrate significant associations between wake-time movement behaviors and mental health problems (17). In fact, wake-time movement compositions appear to explain only a very small amount of the variance in internalizing (3%) and externalizing (8%) problems during this early life stage. This is despite the fact that parent reports indicate 13.1% of TD children and 21.3% of children classified as rDCD in the CATCH cohort score at or above subclinical thresholds for possible mental health problems (23), thus highlighting the importance of identifying effective strategies to mitigate risk beginning at a young age so that these problems do not develop into psychiatric diagnoses. Nevertheless, in light of the complete movement composition explaining minimal variance in indicators of mental health among this sample, it may not be surprising that we found replacing up to 25 min of sedentary time with either LPA or MVPA confers negligible benefits for mental health. Although recent cross-sectional studies have shown movement compositions are positively associated with important physical and cognitive developmental outcomes in the early years (17–19), the benefits for mental health and well-being may not manifest until children are of school age (22). Despite these findings, it is worth noting that accelerometry data does not provide context-specific information about the activities people are engaging in; and therefore, recent advances in machine learning approaches for classifying device-measured movement behaviors into specific activities (e.g., free play, organized sport, cycling) may provide novel insights about the complexity of these relationships (55).

Although five out of the six relationships between movement behaviors and indicators of mental health were not found to be significant, a significant negative association was observed for sedentary time and externalizing problems. One potential explanation for this finding relates to measurement, not only related to the CBCL, but also accelerometry. Within the attention problems subscale (which together with the aggressive behavior subscale make up the externalizing problems scale) are items that may have implications for classification of accelerometer-based movements. For example, one item asks about whether a child cannot sit still. Given that hip worn accelerometers cannot distinguish between sedentary postures (i.e., sitting vs. standing) (56), it is a distinct possibility that sedentary time is misclassified as LPA if a child is constantly fidgeting in their seat or while they stand. For children who score high on measures of attention problems and/or may be diagnosed with ADD/ADHD—a common co-morbidity for children diagnosed with DCD (57)—these instances would serve to strengthen negative associations between sedentary time and externalizing problems. Contrary to this argument, however, is that these children may be expending ≥1 MET and these movement are therefore accurately captured. Moving forward, postural data obtained from wearables that measure posture may help to improve the classification of lower intensity movement behaviors, particularly among individuals with inattention problems.

From a public health standpoint, our findings suggest that movement behavior-related interventions may have limited efficacy when seeking to reduce mental health problems among preschool children. This is not to say that interventions focused on improving movement compositions should be avoided, as they have the potential to provide important benefits for cardiometabolic health and cognitive function (17–19), but rather, that individuals involved in developing health promotion strategies and campaigns for preschool children should be aware that effects for emotional and behavioral outcomes may be negligible. Until researchers begin employing methodologies that provide contextual information about what types of activities preschool children engage in during their sedentary time [i.e., mentally active vs. passive; (58)] or while active, in addition to who they engage in these activities with, we will only have a gross understanding of associations between movement compositions and mental health. Implementing such methodologies with young children may be fraught with challenges though.

Although the present paper addresses gaps in the existing literature, it is not without limitations. First, movement behaviors are traditionally examined using the 24-h paradigm, which also includes sleep. However, data pertaining to sleep duration was not collected in the CATCH study; thus, some of the variance in mental health problems explained by sleep was not captured. Second, the sample consisted of primarily White children living in households with a total income above the poverty line, which may potentially limit the generalizability of our findings to the broader Canadian population, although time spent engaging in each movement behavior was similar to that observed among a nationally representative sample of Canadian preschool children (59). Third, indicators of children's mental health were parent reported given the age of the sample, but evidence indicates parents experiencing mental health problems—mothers in particular—may provide biased reports when asked to assess the mental health of their child (60–62). Future studies would benefit from collaborative partnerships with clinicians who can provide a comprehensive mental health screening for pediatric study participants. Finally, this study involved cross-sectional data, which limits our ability to make causal inferences about the directionality of the relationships between movement compositions and mental health.

In conclusion, we showed that wake-time movement compositions were similar among preschool children classified as TD and rDCD. These findings indicate that differences in sedentary time and physical activity typically observed between these groups likely develop later in childhood. Furthermore, findings revealed that at this young age, the overall composition of wake-time movement behaviors does not appear to play an influential role for children's mental health. While movement behavior-related interventions may have beneficial effects for other important developmental outcomes, interventionists should be aware that the impact on mental health may be trivial.

## Data Availability Statement

The raw data supporting the conclusions of this article will be made available by the authors, without undue reservation.

## Ethics Statement

The studies involving human participants were reviewed and approved by Hamilton Integrated Research Ethics Board. Written informed consent to participate in this study was provided by the participants' legal guardian/next of kin.

## Author Contributions

DB: conceptualization, methodology, writing—original draft, formal analysis, data curation, and visualization. MK: methodology, writing—review and editing, supervision, and funding acquisition. SK-D: writing—review and editing. JC: methodology, writing—review and editing, supervision, and funding acquisition. All authors contributed to the article and approved the submitted version.

## Funding

The Coordination and Activity Tracking in Children (CATCH) study was funded by the Canadian Institutes of Health Research (CIHR Award #: MOP 126015).

## Conflict of Interest

The authors declare that the research was conducted in the absence of any commercial or financial relationships that could be construed as a potential conflict of interest.

## Publisher's Note

All claims expressed in this article are solely those of the authors and do not necessarily represent those of their affiliated organizations, or those of the publisher, the editors and the reviewers. Any product that may be evaluated in this article, or claim that may be made by its manufacturer, is not guaranteed or endorsed by the publisher.
